# Lithium surveillance by community pharmacists and physicians in ambulatory patients: a retrospective cohort study

**DOI:** 10.1007/s11096-022-01420-9

**Published:** 2022-07-13

**Authors:** Jurriaan M. J. L. Brouwer, Arne J. Risselada, Marinka de Wit, Janniek Lubberts, Henrieke Westerhuis, Bennard Doornbos, Hans Mulder

**Affiliations:** 1 Department of Clinical Pharmacy, Wilhelmina Hospital Assen, Mailbox: 30.001, 9400 RA Assen, The Netherlands; 2 GGZ Drenthe Mental Health Services Drenthe, Assen, The Netherlands; 3 Department of Psychiatry, Research School of Behavioral and Cognitive Neurosciences, University of Groningen, University Medical Centre Groningen, Groningen, The Netherlands; 4 Department of Pharmacotherapy, -Epidemiology and -Economics, Department of Pharmacy and Pharmaceutical Sciences, University of Groningen, Groningen, The Netherlands; 5 Lentis Psychiatric Institute, Lentis Research, Groningen, The Netherlands; 6 Dutch Academic Network of Northern Pharmacies (ANNA), Groningen, The Netherlands

**Keywords:** Community pharmacy services, Drug monitoring, Lithium, Pharmacy intervention

## Abstract

**Background:**

Shared care agreements between clinical pharmacists and physicians can improve suboptimal lithium monitoring in in- and outpatient settings. However, it is unknown whether incorporating community pharmacists in such agreements can also improve lithium monitoring in an outpatient setting.

**Aim:**

To assess the necessity for a shared care agreement for lithium monitoring in our region by investigating: intervention rates by community pharmacists and whether those are sufficient; lithium monitoring by physicians in ambulatory patients; the extent of laboratory parameter exchange to community pharmacists.

**Method:**

Patient files of lithium users were surveyed in a retrospective cohort study among 21 community pharmacies in the Northern Netherlands. Outcome was the intervention rate by community pharmacists and whether those were deemed sufficient by an expert panel. Additionally, we investigated both the percentages of patients monitored according to current guidelines and of laboratory parameters exchanged to community pharmacists.

**Results:**

129 patients were included. Interventions were performed in 64.4% (n = 29), 20.8% (n = 5), and 25.0% (n = 1) of initiations, discontinuations, and dosage alterations of drugs interacting with lithium, respectively. The expert panel deemed 40.0% (n = 14) of these interventions as “insufficient”. Physicians monitored 40.3% (n = 52) of the patients according to current guidelines for lithium serum levels and kidney functions combined. Approximately half of the requested laboratory parameters were available to the community pharmacist.

**Conclusion:**

Intervention rates by community pharmacists and lithium monitoring by physicians can be improved. Therefore, a shared care agreement between community pharmacists, clinical pharmacists, and physicians is needed to improve lithium monitoring in ambulatory patients.

**Supplementary Information:**

The online version contains supplementary material available at 10.1007/s11096-022-01420-9.

## Impact Statements

- A shared care agreement might resolve potential barriers impeding interprofessional collaboration and data sharing.
- Training and education for community pharmacists about lithium may improve the pharmacists’ awareness towards lithium monitoring.

## Introduction

Lithium is effective in the prevention of both manic and depressive episodes and the only mood stabilizing drug associated with a lower risk of suicide in patients with bipolar disorder [1, 2]. In addition, lithium can be considered as an option for augmentation therapy in patients with treatment-resistant depression [3]. However, due to its narrow therapeutic window, lithium is associated with severe adverse effects on renal, central nervous system, and thyroid function [4, 5]. Therefore, regular monitoring of lithium serum levels and kidney function is part of national and international clinical practice guidelines (CPGs) [6–8]. Despite that, monitoring of kidney function and lithium serum levels in patients using lithium is suboptimal in daily clinical practice [9–11]. A possible explanation for this is the lack of clarity in who is responsible for monitoring [12, 13]. To improve lithium monitoring, Eagles et al. and Kirkham et al. recommend an integration of primary and secondary care via shared care agreements between physicians. Results showed that the number of patients using lithium being correctly monitored, increased after shared care agreements were implemented [14–16].

Furthermore, several studies in both in- and outpatient settings showed that the involvement of clinical pharmacists in the physicians’ medication policy and monitoring practices resulted in a reduction of potentially inappropriate medication and inadequate blood-test monitoring, and may thus be of added value in a shared care agreement [17–26]. Apart from clinical pharmacists, incorporating community pharmacist-support in these agreements is also needed to improve monitoring practices of lithium in an outpatient setting [27]. Furthermore, involvement of community pharmacists in shared care agreements is facilitated by the Dutch Medicines Act and the Royal Dutch Pharmacists Association (KNMP), who emphasize the availability of necessary laboratory parameter values, such as lithium serum levels and kidney function, to the community pharmacist to ensure safe drug use [28, 29].

However, it is, to our knowledge, unknown to what extent community pharmacists in the Northern Netherlands are currently actively ensuring safe lithium use and whether it could potentially benefit from a shared care agreement between community pharmacists, clinical pharmacists, and physicians. Herein, community pharmacists support physicians in handling drug-drug interactions (DDIs) and notifying them when recent lithium serum levels and/or kidney functions are lacking.

### Aim

Therefore, the aims of this study were to assess the necessity for a shared care agreement in our region by investigating:

- The current intervention rate by community pharmacists regarding DDIs and outdated laboratory parameter values when dispensing lithium to ambulatory patients;
- Whether performed interventions regarding DDIs are deemed sufficient by an expert panel;
- Lithium monitoring by physicians in ambulatory patients using lithium according to the Multidisciplinary Guideline Bipolar Disorders (MGBD) (at least one lithium serum level and kidney function per six months during lithium use) [6].
- The extent of laboratory data exchange to community pharmacists.

### Ethics approval

The study protocol (rTPO 1084) was judged by rTPO Leeuwarden, an independent medical ethics committee. Formal review was waived in October 2019 since participants were not subjected to procedures, nor were they required to follow rules of behavior for this study. Nevertheless, patients were asked to give informed consent, due to the General Data Protection Regulation (GDPR) in Europe.

## Method

### Design and study population

We performed a retrospective cohort study in 21 community pharmacies located throughout two provinces in the Northern Netherlands: Drenthe and Groningen. Patients were eligible for inclusion when they were registered in one of the participating pharmacies and had been using lithium for at least six months prior to the index date (01-01-2019). Eligible patients were asked to participate by their pharmacist by phone, mail, or during a pharmacy visitation.

We defined the observation period as the five year period preceding the index date (01-01-2019), as this period was deemed sufficient for an overview of monitoring practices of lithium by both the physician and community pharmacist.

We defined lithium starters as patients that commenced treatment with lithium (ATC N05AN01) during the observation period. We defined chronic users of lithium as patients that used lithium (ATC N05AN01) before the start of the observation period. Possible lithium dosage forms are lithiumcarbonate IR (200 mg/300 mg/400 mg) or ER tablets (400 mg), lithiumcarbonate IR capsules (100 mg/150 mg/225 mg/300 mg), and lithiumcitrate syrup (34 mg/ml). By default, time periods between two consecutive distributions are one month, whereas this is one week for patients with machine-dispensed sachets which indicate the time of administration.

### Study parameters

Lithium serum levels and kidney functions (eGFR) were defined as laboratory parameters as only these parameters are necessary for the pharmacists for adequate medication monitoring of lithium [28, 29]. Laboratory parameters values of “Certe” were used to assess the monitoring practice of the physician, as “Certe” is the main provider of ambulant diagnostic clinical chemistry in the Northern Netherlands.

The pharmacist can have access to these laboratory parameter values either by using a stand-alone laboratory-pharmacy portal (“Apoview”), in which “Certe” exchanges data with pharmacists after the patients’ consent, or by asking the physician to exchange data for medication safety purposes, see Supplementary Fig. 1. Therefore, laboratory parameter values that were present in “Apoview” and/or the patient files were used to assess the availability of the laboratory parameter values to the community pharmacist.

Based on the MGBD, laboratory parameter values were defined as recent when they were available to the community pharmacist within six months prior to lithium dispensing [6]. Otherwise they were defined as outdated. If no laboratory parameter values were available to the community pharmacist we defined it as absent. We defined lithium serum levels as aberrant when they were lower or higher than 0.4 mmol/L or 1.2 mmol/L, respectively [6]. Kidney function was defined as aberrant when it was lower than 50 ml/min [28, 29].

### Interventions

Based on the MGBD [6] there are two situations in which a pharmacist is expected to intervene before lithium is dispensed:

- Lithium serum levels and/or kidney functions are absent or outdated at the moment of dispensing.
- An interacting drug is initiated, discontinued or altered in dosage.

We regarded lithium surveillance to be adequate when a pharmacist intervened in these two situations. It was agreed upon that if interventions were not registered by the community pharmacist in the patient file we assumed no interventions were performed.

Medication data in the community pharmacies patient files were used to assess the use of lithium and possible DDIs. DDIs with lithium were defined and weighed for clinical relevance by the KNMP using the classification system by van Roon et al. which consists of 6 items (A-F) ranging from a clinically irrelevant effect (A) to death (F) [30]. Data collection was completed in November 2020.

An expert panel was installed to assess if performed interventions, in terms of DDIs, were expedient regarding safe medication use, categorizing it either “sufficient” or “not sufficient”. The expert panel consisted of a psychiatrist, specialized in the treatment of bipolar disorder (B.D.) and a clinical pharmacist / clinical pharmacologist (A.J.R.). Both experts separately assessed the performed interventions in which they received information on the patients’ sex, age, and lithium indication as well as the interacting drugs’ name, the most recent lithium serum level value and kidney function and whether these were available for the community pharmacist, and which interventions were performed. All disagreements between the experts were resolved by consensus.

### Outcome measures

Outcomes were (1) the current intervention rate by community pharmacists regarding DDIs and outdated laboratory parameter values when dispensing lithium to ambulatory patients, (2) whether the performed interventions regarding DDIs were deemed sufficient by an expert panel, (3) the percentage of ambulatory patients that was monitored by their physician according to the MGBD for lithium serum levels and kidney function, and (4) the percentage of laboratory data exchange to community pharmacists.

### Statistical analysis

All statistical analyses were performed with IBM SPSS Statistics version 25.0 for Windows (Armonk, New York, USA). For continuous variables, the means and standard deviations were determined. Medians were presented for continuous variables that were not normally distributed. Frequencies were calculated for categorical variables. The significance level was set at *p* < 0.05.

## Results

### Participants

In total, 415 patients using lithium were identified in the 21 community pharmacies. Of these patients 129 met the inclusion criteria, see Supplementary Fig. 2. Table 1 shows the characteristics of the included patients.

**Table 1:** Characteristics of study population

Variable	Study population (n = 129)	
*Gender*		
Female	74 (57.4%)	
Male	55 (42.6%)	
*Type of user*		
Chronic	91 (70.5%)	
Starter	38 (29.5%)	
*Age (years)*		
Median | Range	60.04 | 23–87	
*Indication lithium*		
Bipolar disorder	61 (47.3%)	
Unipolar disorder	33 (25.6%)	
Unspecified	20 (15.6%)	
Schizophrenic episode	14 (10.9%)	
Cluster headache	1 (0.8%)	
Data exchange Apoview possible^a^	84 (65.1%)	

Study population (n = 129)	Median	Range
Lithium dispensations (No.)^b^	28	4–498
Observation period (months)	58	7–61
Duration of lithium use (months)	268	19–719
*Laboratory parameters*^b^		
Lithium serum level (No.)	17	0–66
Kidney function (No.)	16	0–81

^a^Data exchange possible between pharmacy and laboratory-pharmacy portal (Apoview)

^b^ During the observation period

### Interventions

Lithium was dispensed 6806 times during the observation period. In 46.1% (n = 3136) and 41.3% (n = 2812) of these times, lithium serum levels or kidney functions were outdated, in which in 0.2% (8/3136) and 0.2% (6/2812) interventions were performed by the community pharmacist, respectively. In 50.0% (4/8) of the performed interventions regarding outdated lithium serum levels, the community pharmacists contacted the physician to request a new lithium serum level.

**Fig. 1:**
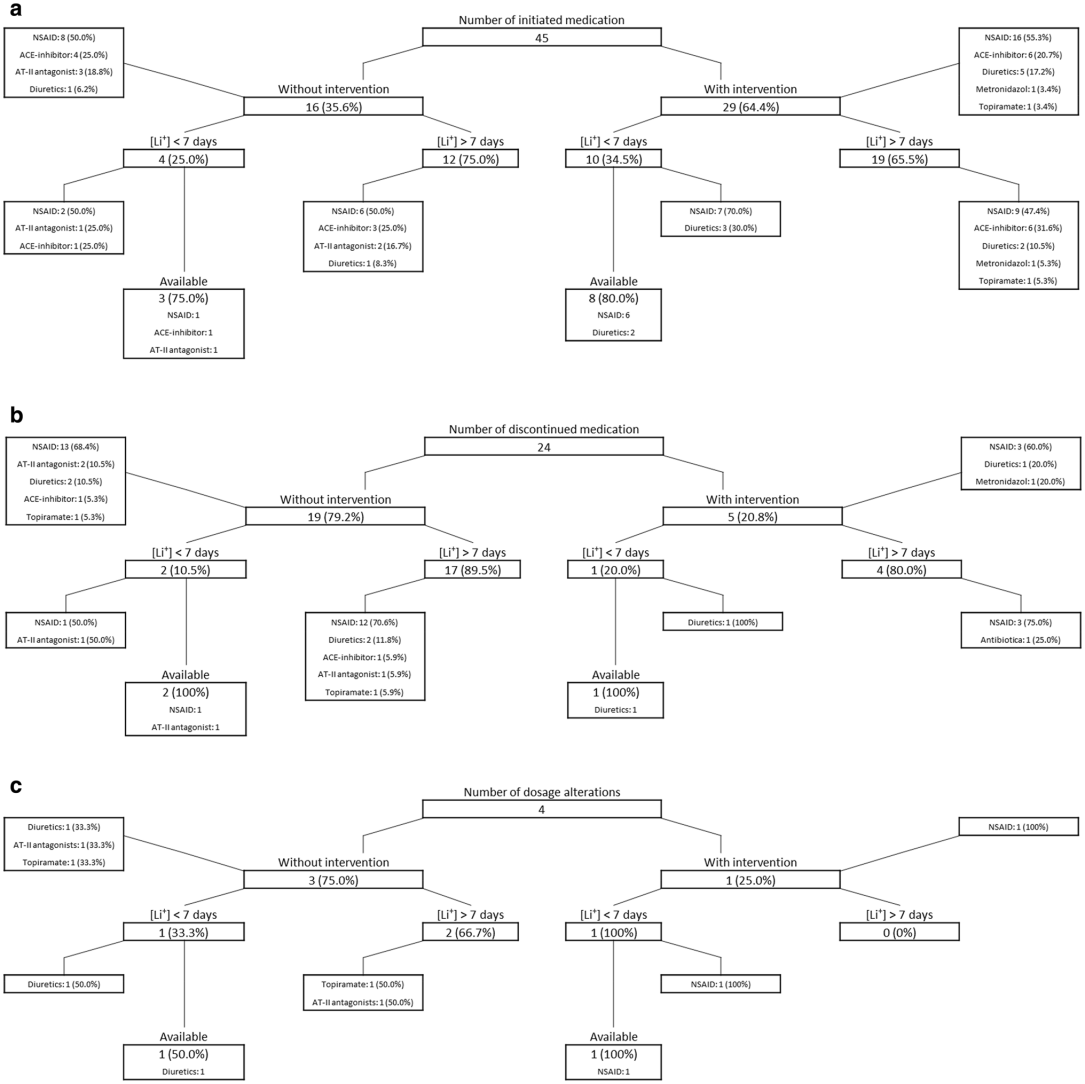
Number of initiated (**a**) and discontinued (**b**) interacting drugs, and interacting drugs that were altered in dosage (**c**), in which interventions were performed and whether lithium serum levels were requested within 7 days (and available to the community pharmacy) after initiation, discontinuation, or dosage alteration of interacting drugs

Figure 1 shows the total number of interacting drugs that were initiated (a), discontinued (b), and altered in dosage (c), in which in 64.4% (n = 29), 20.8% (n = 5), and 25.0% (n = 1) interventions were performed, respectively. The KNMP classified all interacting drug groups as “D”, which is translated to “long lasting (> 7 days) or lasting residual effects or invalidity due to the interaction”.

Table 2 shows the performed interventions by the community pharmacists (n = 35), of which 40.0% (14/35) was classified as “insufficient” by the expert panel. Of these 14 cases, 50.0% (n = 7), 28.6% (n = 4), 14.3% (n = 2), and 7.1% (n = 1) concerned interactions with NSAIDs, ACE-inhibitors, diuretics, and topiramate respectively. In 78.6% (11/14) only the patient was informed about potential adverse effects and/or instructed to inform their physician when interacting drugs were altered in dosage or discontinued. In 3 cases advice was given to the patient, and not to the physician directly, to request a new lithium serum level. Performed interventions which included direct contact with the physician to request new lithium serum levels were classified as “sufficient”.

**Table 2:** An overview of performed interventions when interacting drugs were initiated, discontinued and altered in dosage

Initiated drugs	Performed interventions
NSAIDs	Information regarding toxic effect symptoms: 6 Advising new lithium level request: 1 Handing over an information folder: 5 Substituting interacting medication: 1 Avoid "if necessary" use: 1 Information for patient to instruct treating physician when interacting medication alters/discontinues: 5 Requesting lithium level 3–5 days after start with a potential dosage alteration: 1 Weekly monitoring of lithium levels: 1 Lithium dosage reduction of 50–67%: 1 Requesting new lithium level and kidney function within 7 days after start interacting medication: 1 Request lithium level after start/discontinuing/dose alteration: 1 Contact treating physician, no further intervention: 4
Metronidazol	No intervention, because interaction is irrelevant: 1
ACE-inhibitors	Information regarding toxic effect symptoms: 3 Advising new lithium level request: 1 Handing over an information folder: 2 Requesting lithium level 3–5 days after start with a potential dosage alteration: 1 Information for patient to instruct treating physician when interacting medication alters/discontinues: 1 Lithium dosage reduction of 50–67%: 1 Request lithium level after start/discontinuing/dose alteration: 1 Start lithium after interacting medication treatment: 1
AT-II antagonists	Information regarding toxic effect symptoms: 2 Advising new lithium level request: 3 Handing over an information folder: 3 Requesting lithium level 3–5 days after start with a potential dosage alteration: 1 Advising new lithium level request 3 weeks after starting interacting medication: 1
Topiramate	Weekly monitoring of lithium levels: 1

Discontinued drugs	Performed interventions
NSAIDs	Information regarding toxic effect symptoms: 1 Advising new lithium level request: 2 Contact treating physician, no further intervention: 1
Diuretics	Weekly monitoring of lithium levels: 1 Request lithium level after start/discontinuing/dose alteration: 1
Metronidazol	No intervention, because interaction is irrelevant: 1

Dosage altered drugs	Performed interventions
NSAIDs	Handing over an information folder: 1 Contact treating physician, no further intervention: 1

**Fig. 2:**
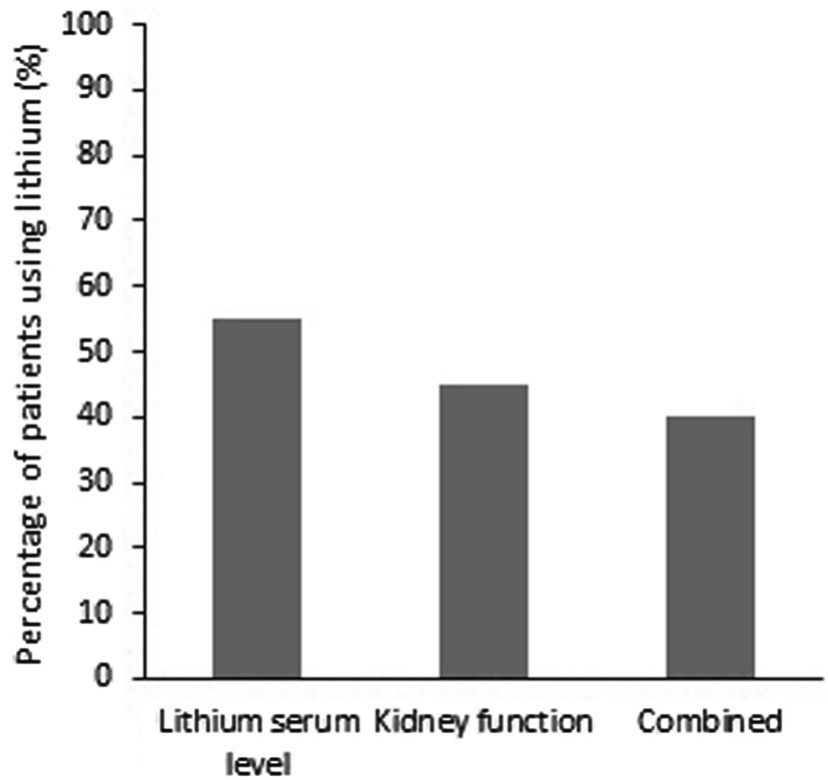
Percentage of patients monitored according to the Multidisciplinary Guideline Bipolar Disorders (MGBD) by physicians [6]

Figure 2 shows the percentages of patients using lithium that were monitored by the physicians according to the MGBD for lithium serum levels (55.0%; 71/129) and kidney function (45.0%; 58/129) separately and combined (40.3%; 52/129).

### Availability of laboratory parameters

A total of 2296 lithium serum levels and 2117 kidney functions were requested by the physicians during the observation period, of which 6.3% (n = 144) and 5.6% (n = 118) were aberrant, respectively. 53.3% (1224/2296) and 51.4% (1088/2117) of the requested lithium serum levels and kidney functions were available to the community pharmacist, respectively.

## Discussion

This study shows that community pharmacists in the Northern Netherlands can substantially improve intervening in (1) DDIs with lithium and (2) outdated lithium serum levels and kidney functions when dispensing lithium to ambulatory patients. Interventions concerning interacting drugs (initiation, discontinuation or dosage alteration) were performed in only 64.4% (29/45), 20.8% (5/24), and 25.0% (1/4) of the cases, respectively. Furthermore, 40.0% (n = 14) of these interventions was deemed “insufficient” by the expert panel. As lithium surveillance is the responsibility of pharmacists in the Dutch health system, these findings indicate an important flaw in the current practices.

Interventions on outdated lithium serum levels and kidney functions prior to lithium dispensing were only performed in 0.2% of the cases, indicating that community pharmacists have the opportunity to signal and communicate shortcomings in the monitoring of lithium serum levels and kidney function to physicians.

Monitoring of lithium serum levels and kidney function by the physician can be improved as well. Only 40.3% (n = 52) of the patients were monitored at least once per six months for lithium serum levels and kidney functions combined.

A few limitations have to be addressed. First, we did not register whether interventions were accepted by the physicians or why community pharmacists did not contact the physician during the majority of interventions. The lack of contact between community pharmacists and physicians could illustrate the current professional relationship between these healthcare professionals treating patients using lithium. Second, there is the potential of information bias; as we surveyed patient files for interventions performed by the community pharmacists, we assumed no interventions were performed if interventions were not registered by the community pharmacist. Therefore, we potentially underestimated the community pharmacists’ intervention rates. Finally, we realize that our current results and view on the community pharmacists’ potential future role herein, might be considered as being specific for the situation in the (Northern part of the) Netherlands, and therefore do not reflect the situation regarding lithium monitoring in other countries. However, research [9, 31–33] has shown that lithium monitoring is also suboptimal in the UK and in Japan. Therefore we believe that our results can be, at least to some degree, extrapolated to other countries and that our view on the community and clinical pharmacists’ potential future role in lithium monitoring can serve as an example in how to potentially improve lithium monitoring in an outpatient setting.

### Drug-drug interactions

Several potential factors may contribute to the low number of interventions during DDIs with lithium in this study. In situations where lithium serum levels were available within 7 days after a drug-drug interaction (6/38), the community pharmacist may have concluded that the physician already acted on the event and an intervention would therefore not be necessary.

Another reason may be the absence of a signal by the medication safety system when interacting drugs are discontinued. In these cases the community pharmacist is not notified about the possible consequences discontinuation of the interacting drug might have on the lithium serum level, resulting in the absence of an intervention.

40.0% (14/35) of the performed interventions were deemed “insufficient” by the expert panel, which mostly involved interventions in which, for example, only the patient was informed about potential adverse effects. We assume the information was solely given to the patient as the community pharmacist could state that the physician was contacted. In this way, the responsibility in follow-up shifts from the physician and community pharmacist towards the patient. We believe that a more active and supportive role by the community pharmacist towards the patient and their physician is needed in order to improve safe lithium use.

Although community pharmacists are willing to pursue a more active role in their care towards their patients with severe mental illness, they might not think it is their task to intervene or lack confidence for consulting the physician [34, 35]. Several other factors may contribute to the low number of perceived moments of contact between the community pharmacist and the physician. Bollen et al. [36] investigated factors that influenced interprofessional collaboration in the community setting. Next to i.e. confidence in the collaborations’ benefits and good communication, they found that close proximity between professions positively affects collaboration, medication management [17, 37, 38], and monitoring [39].On the other hand, factors impeding collaboration are i.e. lack of clarity regarding each other’s roles and responsibilities, lack of trust, respect, and time [36]. Therefore, co-location, creating awareness of each other’s roles/capabilities, and co-education and regular meetings may be options to improve interprofessional collaboration between community pharmacists and physicians [40, 41]. Herein, the clinical pharmacist may be of assistance in bridging this gap between these healthcare professionals.

### Laboratory parameter values

The Dutch Medicines Act and the KNMP emphasize the availability of necessary laboratory parameter values to the pharmacist [28, 29]. However, only 53.3% and 51.4% of the lithium serum levels and kidney functions were available to the community pharmacist, respectively. Furthermore, the low intervention rates on laboratory parameter values when dispensing lithium indicate an inactive role of the community pharmacists towards acquiring recent laboratory parameter values.

A possible explanation for the low availability of kidney functions in our study is the low percentage of impaired kidney functions (5.6%; 118/2117), as in the Netherlands, prescribers are legally obligated to share impaired kidney functions with the pharmacists [28, 29]. A second possible explanation might be the patients’ relatively low authorization rate for the pharmacists to access laboratory parameter values (65.1%; 84/129).

Possible options to improve availability of laboratory parameters to community pharmacists is to stimulate patients to grant their pharmacist access to necessary laboratory parameter values and/or a medical pharmaceutical decision rule for lithium use, which was introduced in 2020 by the KNMP for the pharmacy chain BENU community pharmacies [42]. This decision rule stated that kidney functions (when impaired) and lithium serum levels should be monitored every six months. This might improve the community pharmacists’ alertness and assertiveness in retrieving up-to-date laboratory parameters.

Another possible option is when community pharmacists start monitoring these laboratory parameters themselves, as community pharmacists are able to monitor kidney functions by means of point-of-care testing (PoCT) [43]. Monitoring lithium serum levels by means of PoCT are also available. Therefore, one might speculate that community pharmacists are also able to monitor lithium serum levels [44]. However, whether in the future this responsibility might lie with the community pharmacist, is up for debate, as physicians need to agree with this and a possible lack of comfort and confidence of the community pharmacist in performing PoCT of lithium serum levels might be present [34, 35].

### Monitoring practices by physicians

Monitoring practices of lithium serum levels and kidney function among patients using lithium is suboptimal. Our study presents similar findings compared with studies conducted among ambulatory patients using lithium [9–11]: 55.0% (71/129) and 45.0% (58/129) of the patients was not monitored according to the guideline for lithium serum levels or kidney functions, respectively.

Although the request of lithium serum levels by community pharmacists is up for debate, we do believe that an improvement in the management of lithium therapy in an outpatient setting may be achieved by a shared care agreement between community pharmacists, clinical pharmacists, and physicians. Herein community pharmacists support physicians in handling DDIs and notifying them when recent lithium serum levels and/or kidney functions are lacking.

Therefore, it is currently being investigated in the Northern Netherlands, whether an improvement in monitoring practices is feasible in an outpatient setting by means of training, education, and a shared care agreement between community pharmacists, clinical pharmacists, and physicians.

## Conclusion

This study indicates that community pharmacists can intervene more actively during lithium dispensing when DDIs with lithium are concerned and/or necessary laboratory parameter values are outdated, whereas physicians can improve lithium monitoring. Therefore we currently conduct a prospective study to investigate whether a shared care agreement between community pharmacists, clinical pharmacists, and physicians might improve lithium monitoring in ambulatory patients.

## Supplementary Information

Below is the link to the electronic supplementary material.

Supplementary file1 (PDF 210 kb)

Supplementary file2 (PDF 159 kb)
