# Supplementary material for: Lithium surveillance by community pharmacists and physicians in ambulatory patients: a retrospective cohort study
Source: Int J Clin Pharm. 2022 Jul 13;44(4):975–84. doi: 10.1007/s11096-022-01420-9 (PMC9393139; doi:10.1007/s11096-022-01420-9)
Supplement: Supplementary file 1 — Supplementary file1 (PDF 210 kb) [file 11096_2022_1420_MOESM1_ESM.pdf]

Title: Lithium surveillance by community pharmacists and physicians in ambulatory patients; a retrospective cohort study

Journal: International Journal of Clinical Pharmacy

Authors: Jurriaan M.J.L. Brouwer, Pharm.D.[1,2,3], Arne J. Risselada, Pharm.D., Ph.D.[1], Marinka de Wit, Pharm.D.[4], Janniek Lubberts, Pharm.D.[4], Henrieke Westerhuis, Pharm.D.[4], Bennard Doornbos, M.D., Ph.D.[5], Hans Mulder, Pharm.D., Ph.D.[1,6]

Affiliations: 1. Department of Clinical Pharmacy, Wilhelmina Hospital Assen, Assen, The Netherlands 2. GGZ Drenthe Mental Health Services Drenthe, Assen, The Netherlands 3. Department of Psychiatry, Research School of Behavioral and Cognitive Neurosciences, University of Groningen, University Medical Centre Groningen, Groningen, The Netherlands 4. Department of Pharmacotherapy, -Epidemiology & -Economics, Department of Pharmacy and Pharmaceutical Sciences, University of Groningen, Groningen, Groningen, The Netherlands 5. Lentis Psychiatric Institute, Lentis Research, Groningen, The Netherlands 6. Dutch Academic Network of Northern Pharmacies (ANNA), Groningen, The Netherlands

E-mail corresponding author: hans.mulder@wza.nl

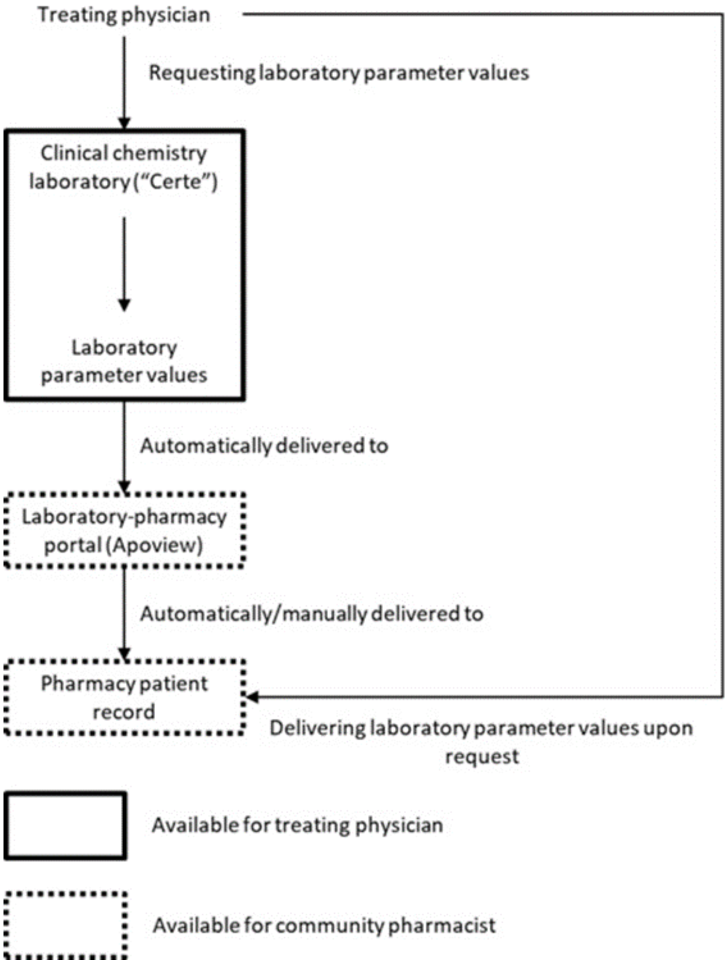

Supplementary Fig. 1 *Process of the request, determination, and availability of laboratory parameter values*
